# Evaluating the Clinical Validity of the Meal Rounds Observation Form for Assessing Safe Food Intake in Patients with Dysphagia: A Multicenter Prospective Study

**DOI:** 10.3390/nu18081226

**Published:** 2026-04-14

**Authors:** Mitsuko Shimizu, Junko Fujitani, Ichiro Fujishima, Takehiro Karaho, Takeshi Kikutani, Yutaka Watanabe, Seiko Shibata, Yasushi Fujimoto, Mitsuyoshi Yoshida

**Affiliations:** 1 Speech-Language Pathology Division, Department of Rehabilitation, Saitama Prefectural Rehabilitation Center, 148-1 Nishikaiduka, Ageo-shi 362-8567, Japan; 2 Department of Rehabilitation, Japan Institute for Health Security, National Center for Global Health and Medicine, 1-21-1 Toyama, Shinjuku-ku, Tokyo 162-8655, Japan; fujitani.j@jihs.go.jp; 3 Hamamatsu City Rehabilitation Hospital, 1-6-1 Wago-Kita, Chuo-ku, Hamamatsu-shi 433-8511, Japan; ifujishima@sis.seirei.or.jp; 4 Jindai Ear-Nose-Throat Clinic, 2-23-5-302, Jindaiji-Higashimachi, Chofu-shi 182-0011, Japan; tkaraho@jindai-jibika.com; 5 Division of Clinical Oral Rehabilitation, Graduate School of Life Dentistry at Tokyo, Nippon Dental University, 4-44-19 Higashi-cho, Koganei-shi 184-0011, Japan; kikutani@tky.ndu.ac.jp; 6 Gerodontology, Department of Oral Health Science, Faculty of Dental Medicine, Hokkaido University, Nishi-7, Kita-13, Kita-ku, Sapporo 060-8586, Japan; ywata@den.hokudai.ac.jp; 7 Department of Rehabilitation Medicine, School of Medicine, Fujita Health University, 1-98 Dengakugakubo, Kutsukake-cho, Toyoake-shi 470-1192, Japan; sshibata@fujita-hu.ac.jp; 8 Department of Otolaryngology-Head and Neck Surgery, Aichi Medical University, 1-1 Yazakokarimata, Nagakute-shi 480-1195, Japan; yasushif@aichi-med-u.ac.jp; 9 Department of Dentistry and Oral-Maxillofacial Surgery, School of Medicine, Fujita Health University, 1-98 Dengakugakubo, Kutsukake-cho, Toyoake-shi 470-1192, Japan; mitsuyoshi.yoshida@fujita-hu.ac.jp

**Keywords:** swallowing, dysphagia, meal rounds, videofluoroscopic examination of swallowing (VF), videoendoscopic examination of swallowing (VE)

## Abstract

**Background/Objectives:** Providing an appropriate diet to older adults with dysphagia can prevent aspiration, choking, and nutritional deficiencies and help preserve their quality of life. Therefore, assessments for determining the appropriateness of food types are required. This multicenter study aimed to determine the reliability and validity of the Meal Rounds Observation Form (MROF), which was developed to identify food forms that can be safely consumed by older adults with dysphagia. **Methods:** We analyzed 532 food–texture observations obtained from 155 participants (114 men and 41 women). The reliability and validity of the MROF were compared with those of videofluoroscopic (VF) or videoendoscopic (VE) examinations of swallowing. **Results:** The food-form categories were water (108 pairs), 0j (54 pairs), 0t (118 pairs), 1j (20 pairs), 2-1 (28 pairs), 2-2 (37 pairs), 3 (68 pairs), 4 (67 pairs), and normal food (32 pairs) based on JDD 2021 codes. The AUC was lowest for the water (0.568) category and highest for food forms requiring chewing, such as those of the 4 and normal food (0.678) categories. The sensitivity and specificity of the Gugging Swallowing Screen were 60.1% and 69.1%, respectively (*p* < 0.001). The agreement between the Gugging Swallowing Screen and the MROF evaluation for food types requiring mastication was 73.2%. Logistic regression analysis revealed asymmetric movement of the corners of the mouth and coughing as important indicators when evaluating food types requiring mastication. **Conclusions:** The MROF is useful for determining food intake safety when VF or VE tests cannot be performed in medical and nursing care settings and can guide clinical decision-making. However, caution is required in applying it clinically because of its relatively low specificity.

## 1. Introduction

The number of older adults with dysphagia is increasing in the super-aging society of Japan. A recent study by Igarashi et al. [1] revealed that 25.1% of healthy older adults in the community and 53.8% of residents of long-term care facilities have dysphagia. Providing an appropriate diet to patients with dysphagia may prevent aspiration, choking, and nutritional deficiencies and maintain quality of life [2].

In Japan, the Japanese Society for Swallowing Rehabilitation published the “Japanese Dysphagia Diet 2013” (JDD2013) by the Swallowing Diet Committee in 2013 [3] to classify food forms. The diet was revised in 2021 (JDD2021); however, the core principles remain unchanged [4]. A professional assessment is required to determine the appropriateness of food texture for patients with dysphagia in medical and nursing care settings.

Videofluoroscopic (VF) or videoendoscopic (VE) examination of swallowing is important for assessing feeding and swallowing functions and determining appropriate food types. However, these tests are not available in all healthcare facilities, nursing homes, or home settings. Therefore, meal-rounds observation, a method of selecting appropriate food types based on dietary observations by multiple professionals, is recommended [5,6]. Unfortunately, there are currently no guidelines for this approach.

Evaluation methods must be developed to select appropriate food types. Previous screening tests for masticatory function have directly examined the mixing state of two differently colored chewing gums or paraffin waxes immediately after chewing [7,8]. However, both methods only evaluate masticatory efficiency. The Mann Assessment of Swallowing Ability (MASA) [9,10] was developed to screen swallowing function in patients with stroke, but it is also applicable to non-stroke cases. Its results have been compared with those of VF examinations using thickened liquids and semi-solid foods, demonstrating its validity. MASA consists of 24 observation and evaluation items covering the oral and pharyngeal phases to assess a patient’s swallowing function. The evaluation criteria are numerous and highly specialized, making them difficult to use. Therefore, this method can only be performed by a speech–language pathologist.

In contrast, the Gugging Swallowing Screen (GUSS) [11,12] evaluates the intake of test meals at three levels of difficulty and suggests appropriate food types based on the total score. This method can be performed by any healthcare professional. However, its validity has been tested with and without aspiration for liquid and semi-solid diets using VE, but not for diets requiring mastication.

Therefore, the purpose of this study was to develop a dietary observation form that includes items for evaluating foods requiring mastication and determines their reliability and validity relative to VF and VE evaluation.

## 2. Materials and Methods

This multicenter, prospective, observational study involving 17 hospitals was approved by the Ethics Review Committee of the National Center for Global Health and Medicine through a centralized review process (approval number NCGM-G-003162-00). Eligible participants were adults (≥20 years old) who were diagnosed with dysphagia affecting oral intake and underwent swallowing evaluation with VF or VE at one of the participating institutions between 28 December 2018 and 30 March 2020. Written informed consent was obtained from all participants before enrollment. Individuals younger than 20 years were excluded from this study. This study was approved by the Institutional Ethics Committee of the National Center for Global Health and Medicine (protocol code NCGM-G-003162-00).

To develop the Meal Rounds Observation Form (MROF), the research team was subgrouped into the literature review and clinical survey teams. The literature review team conducted a comprehensive analysis of previous studies on dysphagia assessment and food texture selection. Simultaneously, the clinical survey team conducted a questionnaire-based study in collaboration with eight professional organizations involved in the care of individuals with dysphagia. Certified specialists recognized by these organizations were invited to participate, and 625 valid responses were obtained. These responses were analyzed to identify the key clinical perspectives and decision-making criteria for recommending appropriate food textures. Based on the findings of both teams, the research group conducted a series of discussions and identified nine core observational items. These items were incorporated into the MROF, as listed in Table 1. Of these nine items, swallowing (2), coughing (3), drooling (5), and voice change (6) are the common items evaluated by the GUSS. Therefore, it is possible to calculate the GUSS results based on these findings.

The definitions and evaluation criteria were as follows: (1) Mastication was estimated by assessing the asymmetric movement of the corners of the mouth based on appearance. (2) Swallowing was evaluated for the ability to swallow and for delayed swallowing reflex attraction. (3) The patient was observed before, during, and after swallowing to check for a light, small, or hoarse cough. (4) Cervical auscultation was performed to check for abnormal swallowing and post-swallowing breathing sounds: abnormal sounds such as long swallowing sounds; weak swallowing sounds; bubbling sounds during swallowing; sputum sounds associated with coughing; wet, congested sounds immediately after swallowing; gargling sounds; and liquid vibrating sounds were evaluated [13]. (5) Drooling was evaluated based on the presence or absence of saliva flowing at the moment the participant placed food in the mouth, during chewing, and until swallowing. (6) Voice changes were observed and evaluated by having the participant say “ahh” after swallowing, which sounds like wet hoarseness. (7) Respiration was evaluated by observing changes during meals, particularly during shallow and fast breathing after swallowing. (8) Oral residues were evaluated by observing the residues in the mouth after swallowing. Residues were classified into one of two levels depending on their quantity. (9) The clearance of oral residues was assessed by examining whether the residues were removed by gargling.

A preliminary evaluation was conducted to ensure the reliability of the MROF before its use in the main study. Forty-three raters participated in the inter-rater agreement assessment. The analysis revealed a Cohen’s κ coefficient of 0.73 and a Cronbach’s α coefficient of 0.72, indicating acceptable levels of inter-rater reliability and internal consistency.

In this multicenter study, the MROF was implemented at 14 hospitals. For each patient scheduled to undergo a swallowing evaluation with VF or VE, the MROF was completed in advance by a nurse or speech-language pathologist at the respective institution. To ensure consistency across facilities, standardized criteria were used to evaluate each food texture.

The VF test was performed according to the 2014 guidelines issued by the Medical Review Committee of the Japanese Society for Swallowing Rehabilitation, titled “Procedures for Videofluoroscopic Evaluation of Swallowing (Detailed Version)” [14], and with the Penetration–Aspiration Scale (PASS) [15]. The VE test was conducted following the 2014 Procedures for Swallowing Evaluation by Videoendoscopy by the same committee, and patient evaluation was based on the Hyodo scoring system [16,17].

An experienced dysphagia specialist at each facility, blinded to the MROF results, made an overall clinical judgment regarding the safety of oral intake of each food texture based on the VF or VE test findings. When both the VF and VE tests were conducted, the VF test results were given priority. Additionally, when a food item was tested multiple times, the worst result was used for evaluation.

Food texture classification was coded according to the Japanese Dysphagia Diet 2021 (JDD2021) developed by the Japan Society of Dysphagia Rehabilitation (JSDR) Dysphagia Diet Committee. The codes used in this study are as follows: (a) 0j: homogeneous, adherent, cohesive, firm jelly food that can be scooped into slices; (b) 0t: homogeneous, adherent, cohesive, firm, and thickened water; (c) 1j: homogeneous, adherent, cohesive, firm, water-releasing jelly, pudding, or mousse-like textured food; (d) 2: homogeneous pureed, paste-like, or blended food that is non-sticky and forms a cohesive bolus easily; (e) 3: food with a defined shape that can be easily mashed between the tongue and palate, facilitates bolus formation and transfer, and is prepared to prevent disintegration in the pharynx to make it easier to swallow; and (f) 4: food that requires mastication using teeth or an alveolar ridge but can be cut with a fork or spoon (the food does not easily fall apart and is not prone to sticking).

### Statistical Analysis

For each food form in each case, the overall judgment of safety was compared with the MROF and VF or VE findings. The sensitivity and specificity values were calculated using a 2 × 2 table to examine the validity of the MROF. In addition, we calculated the AUC using the ROC curve. The scores for the GUSS were compared with those of the MROF. For foods requiring chewing, binomial logistic analysis was used to examine items that significantly influenced the determination of safety. IBM SPSS Ver. 26 (IBM, Tokyo, Japan) was used for data analysis. Statistical significance was set at *p* < 0.05.

## 3. Results

The evaluation included 532 data points from 155 participants (114 men and 41 women; median age, 77 years). The participants’ underlying conditions were cerebrovascular disease (128 patients), neurodegenerative disease (20 patients), pneumonia (4 patients), and heart disease (3 patients). The participants underwent an observational evaluation using the MROF and VF or VE tests for each food category at 18 collaborating institutions. The food-form categories were water (108 pairs), 0j (54 pairs), 0t (118 pairs), 1j (20 pairs), 2-1 (28 pairs), 2-2 (37 pairs), 3 (68 pairs), 4 (67 pairs), and normal food (32 pairs) based on JDD 2021 codes.

### 3.1. Agreement on the Ratings for Each Form of Food

Table 2 shows the consistency between the MROF and VF or VE results for each food type. Of the 108 pairs for water, the correctly classified proportion was 0.67. The sensitivity and specificity of this agreement for safe drinking water were 76.5% and 37.0%, respectively, and the AUC was 0.568.

For thickened water, corresponding to code 0t, 87 of the 118 pairs (73.7%) were correctly classified. The sensitivity and specificity for drinking thickened water were 77.9% and 42.9%, respectively, and the AUC was 0.604.

For jellies corresponding to 0j and 1j, the correctly classified proportion was 0.96. The sensitivity and specificity for jelly safety were 98.6% and 33.3%, respectively, and the AUC was 0.660.

For foods that did not require chewing and could easily form food clumps in the oral cavity, such as pastes that fall under codes 2-1, 2-2, and 3, 122 of the 133 pairs (91.7%) were correctly classified. The sensitivity and specificity for safe consumption of paste and easily crushable foods were 94.4% and 42.9%, respectively, and the AUC was 0.634.

For regular foods and those requiring mastication (code 4), 77 of 99 pairs (77.8%) were correctly classified. The sensitivity and specificity for the ability to consume foods that required mastication were 80.0% and 55.6%, respectively, and the AUC was 0.678.

### 3.2. Examination of Observation Items Related to the Safety of Mastication

The observed items related to the safety of ingesting food requiring mastication. The food items were examined using a binomial logistic analysis with the scores of the nine observed assessment items as the independent variables and the VF or VE results as the dependent variables. The significant items were “lateral asymmetric movement of the corners of the mouth (coefficient)” and “cough” (Table 3).

### 3.3. Comparison of GUSS and MROF Evaluations

The overall assessment of the oral availability of all food forms revealed agreement (80.8%) between the MROF and test results for 430 of 530 cases: for 7.7% of cases, the MROF results were more lenient than the test results (acceptable according to the MROF, but not during inspection); for 11.5% of the time, the results were more severe (not acceptable according to the MROF, but acceptable during inspection). The sensitivity and specificity values of the MROF were 86.9% and 39.7%, respectively (*p* < 0.001). Based on the GUSS criteria, evaluation of the data for the four items—swallowing, choking, drooling, and voice quality changes—revealed that the assessments were consistent in 326 cases and inconsistent in 206 cases. The agreement obtained using the GUSS was 60.1% for sensitivity and 69.1% for specificity (*p* < 0.001) (Table 4).

## 4. Discussion

The purpose of this study was to examine the clinical reliability and validity of the MROF created to select appropriate food forms for older adults with dysphagia. Therefore, observation ratings and test results based on the VF or VE test were compared for various food types according to JDD 2021. The AUC values for all dietary food categories except for water fall within the range of 0.60 ≤ AUC < 0.70, indicating a certain level of accuracy. Therefore, the MROF developed in this study appears to be a useful tool for evaluating food-form improvements during meal observation.

Unfortunately, the agreement between the MROF and VF or VE test results regarding water swallowing was not particularly high, with an AUC of only 0.568. One possible reason for this is the presence of factors that cannot be detected by screening tests, such as silent aspiration. This is a challenge faced by all screening tests, and as previous reports have shown, it is necessary to assess patients using a combination of tests [17]. On the other hand, the sensitivity and specificity increased for 0t foods with added viscosity, as previously described [18,19].

The degree of agreement between the MROF and VF or VE test results exceeded 90% for jelly-like foods (0j and 1j) and paste-like and easily crushed foods (2-1, 2-2, and 3). This is attributed to the fact that the study participants were capable of oral intake, and many were able to consume these types of foods safely [20]. However, a few individuals were unable to consume these foods safely, resulting in a low specificity; therefore, further investigation is required to determine whether the MROF can be used in more severely ill patients.

Considering the quality of life of facility residents and other patients, providing meals similar to normal meals is beneficial. Therefore, we examined the items that are important for the safe consumption of foods requiring mastication. The results show that the asymmetric movement of the corners of the mouth and cough are important factors that may be helpful in selecting the type of food that requires mastication. The asymmetric movement of the corners of the mouth and rotational movement of the mandible are closely correlated with tongue movement during mastication. A previous study examining VE results and other observations has shown that tongue movements are closely related to the shape of the food mass [21]. The rating of this study was acceptable for the MROF and VF test when the movement of the corners of the mouth was asymmetric. This suggests that asymmetric movement of the corners of the mouth is associated with higher overall function of oral intake. The Saku Saku Test, developed for a similar purpose, compares the relationship between mandibular movements and the ability to form food masses through mastication [22]. The Saku Saku Test was found to have sensitivities between 45.0% and 63.6% and specificities between 90.6% and 81.8% for food lump formation and the ability to ingest food requiring mastication, respectively. The MROF has a sensitivity of 80.0% and a specificity of 55.6%, demonstrating comparable discriminatory ability. Therefore, the ability to safely ingest foods requiring mastication may be determined by the usual dietary observations of the patient without the need to use special foods.

We also compared the existing GUSS observation rating scale [11,12] with the newly developed nine-item observation rating scale. The results showed that the findings of both tests had statistically significant correlations with the safety of certain foods for oral intake. The GUSS was less sensitive and more specific and determined more cases with an inability to swallow (approximately three times more than the MROF and four times more than the VF or VE test). The GUSS may therefore be too cautious about whether oral intake is possible. As the MROF encompasses all four components of the GUSS, we believe that whilst these four components should be prioritized when risk is a key consideration, the MROF can be effectively utilized to guide the selection of better food options for oral consumption in both institutional and home settings.

Furthermore, an international dietary guideline, “The International Dysphagia Diet Standardization Initiative (IDDSI),” is currently being developed [23], and a comprehensive map between the IDDSI and JDD2021 classifications has also been produced [24]. We plan to examine the relationship between the IDDSI and MROF in the future.

This study has several limitations. It was a multicenter study, and there may have been differences in the evaluations of the raters at each site. The present study describes the unification of the evaluation methods but does not confirm the degree of agreement among the raters. In a multicenter study, prior training via video or other means may be necessary to improve evaluation accuracy. Moreover, the specificity of the MROF is not very high, and the misclassifications observed in some cases suggest that its predictive ability is limited. In particular, the fact that this study did not include patients with severe dysphagia is likely to have reduced the specificity. Consequently, further investigation is required to determine whether the MROF is suitable for use in patients with severe dysphagia. However, we conclude that the MROF can be used to determine whether foods that require mastication can be consumed, which was the main objective of this study. As part of our efforts to promote the wider adoption of the MROF, we created several training videos demonstrating meal observations, which are available online through a dedicated website: https://www.hosp.jihs.go.jp/s027/202010_guideline_development.html (accessed on 10 February 2026).

## 5. Conclusions

This multicenter study aimed to determine the reliability and validity of the Meal Rounds Observation Form (MROF). The results of this study indicate that the MROF is a useful tool for determining the safety of foods for older adults with dysphagia when VF and/or VE testing cannot be performed. Therefore, the MROF may be useful in daily clinical practice because it can be used to evaluate foods that require chewing.

## Figures and Tables

**Table 1:** Meal Rounds Observation Form.

Type of Food (JDD2021)	Code	Code	Code	Code
1. Laterally asymmetric movement of the corners of the mouth (asymmetry)				
Yes	□	□	□	□
No	□	□	□	□
2. Swallowing				
Possible with delay	□	□	□	□
Possible	□	□	□	□
3. Coughing				
Yes	□	□	□	□
No	□	□	□	□
4. Cervical auscultation (auscultation)				
Abnormal sound	□	□	□	□
No abnormal sound	□	□	□	□
5. Drooling				
Yes	□	□	□	□
No	□	□	□	□
6. Voice change				
Yes	□	□	□	□
No	□	□	□	□
7. Respiration observation (respiration)				
Shallow and fast	□	□	□	□
No abnormality	□	□	□	□
8. Oral residue				
Yes	□	□	□	□
Small amount	□	□	□	□
No	□	□	□	□
9. Clearance of oral residue by gargling (clearance)				
Cannot gargle to clear residue	□	□	□	□
Inadequate gargling	□	□	□	□
Can gargle	□	□	□	□

JDD2021: Japanese Dysphagia Diet 2021.

**Table 2:** Percentage of agreement between the MROF and VF or VE test results for each food item.

Judging Whether the Food Items Are Safe to Eat			VF·VE Test	
			Yes	No
MROF	Water	Yes	62	17
			57.4%	15.7%
		No	19	10
			17.60%	9.30%
	0j	Yes	53	1
			98.1%	1.9%
		No	0	0
			0%	0%
	0t	Yes	81	8
			68.6%	6.8%
		No	23	6
			19.5%	5.1%
	1j	Yes	17	1
			85.0%	5.0%
		No	1	1
			5.0%	5.0%
	2-1	Yes	23	3
			82.1%	10.7%
		No	2	0
			7.1%	0%
	2-2	Yes	30	2
			81.1%	5.4%
		No	2	3
			5.4%	8.1%
	3	Yes	66	2
			97.1%	2.9%
		No	0	0
			0%	0%
	4	Yes	54	4
			80.6%	6.0%
		No	8	1
			11.9%	1.5%
	Normal	Yes	18	0
	food		56.3%	0%
		No	10	4
			31.3%	12.5%

**Table 3:** Items of the Meal Rounds Observation Form (MROF) associated with the safety of intake of food requiring mastication (binomial logistic analysis).

Items	B	SE	*p*-Value	95%CI		VIF
1. Asymmetry	0.749	0.283	0.008	1.214	3.684	1.095
2. Swallow	0.317	0.31	0.307	0.747	2.522	1.122
3. Cough	1.939	0.327	0.000	3.665	13.188	1.135
4. Auscultation	0.431	0.443	0.330	0.646	3.663	2.756
5. Drooling	−0.165	0.58	0.776	0.272	2.641	1.120
6. Voice change	−0.193	0.407	0.636	0.371	1.832	2.571
7. Respiration	0.98	0.527	0.063	0.948	7.487	1.251
8. Oral residue	0.498	0.331	0.132	0.86	3.149	1.133
9. Clearance	−0.522	0.488	0.285	0.228	1.544	1.174

**Table 4:** Comparison of the Meal Rounds Observation Form (MROF) and GUSS results with the VE/VF test results for all food forms.

Judging Whether the Food Items Are Safe to Eat		VF·VE Test		Total
		Yes	No	
MROF	Yes	403 (75.8%)	41 (7.7%)	444 (83.5%)
	No	61 (11.5%)	27 (5.0%)	88 (16.5%)
GUSS	Yes	279 (52.4%)	21 (4.0%)	300 (56.4%)
	No	185 (34.8%)	47 (8.8%)	232 (43.6%)
Total		464 (87.2%)	68 (12.8%)	532 (100%)

## Data Availability

The original contributions presented in this study are included in the article. Further inquiries can be directed to the corresponding author.

## Abbreviations

The following abbreviations are used in this manuscript:

GUSS	Gugging Swallowing Screen
JSDR	Japan Society of Dysphagia Rehabilitation
MASA	Mann Assessment of Swallowing Ability
MROF	Meal Rounds Observation Form
PASS	Penetration–Aspiration Scale
QOL	Quality of Life
VE	Videoendoscopic Examination of Swallowing
VF	Videofluoroscopic Examination of Swallowing
IDDSI	International Dysphagia Diet Standardization Initiative
